# Can we predict cognitive decline after initial diagnosis of multiple sclerosis? Results from the German National early MS cohort (KKNMS)

**DOI:** 10.1007/s00415-018-9142-y

**Published:** 2018-12-04

**Authors:** Andreas Johnen, Paul-Christian Bürkner, Nils C. Landmeyer, Björn Ambrosius, Pasquale Calabrese, Jeremias Motte, Nicole Hessler, Gisela Antony, Inke R. König, Luisa Klotz, Muna-Miriam Hoshi, Lilian Aly, Sergiu Groppa, Felix Luessi, Friedemann Paul, Björn Tackenberg, Florian Then Bergh, Tania Kümpfel, Hayrettin Tumani, Martin Stangel, Frank Weber, Antonios Bayas, Brigitte Wildemann, Christoph Heesen, Uwe K. Zettl, Frauke Zipp, Bernhard Hemmer, Sven G. Meuth, Ralf Gold, Heinz Wiendl, Anke Salmen, German Competence Network Multiple Sclerosis (KKNMS), Seray Demir, Christoph Schröder, Lisa A. Voithenleitner, Achim Berthele, Sarah Haars, Sandra Nischwitz, Matthias J. Knop, Susanne Rothacher, Jana Pöttgen, Clemens Warnke, Ralf A. Linker, Ulf Ziemann

**Affiliations:** 1 Department of Neurology, University Hospital Münster, Westfälische-Wilhelms-University Münster, Münster, Germany; 2 Department of Statistics, Faculty of Psychology, Westfälische-Wilhelms-University, Münster, Germany; 3 Department of Neurology, St. Josef-Hospital, Ruhr-University Bochum, Bochum, Germany; 4 Department of Neuropsychology and Behavioral Neurology, University of Basel, Basel, Switzerland; 5 Institute of Medical Biometry and Statistics, University of Lübeck, University Hospital Schleswig-Holstein, Campus Lübeck, Lübeck, Germany; 6 Central Information Office (CIO), Philipps-University Marburg, Marburg, Germany; 7 Department of Neurology, Klinikum rechts der Isar, Technical University of Munich, Munich, Germany; 8 Munich Cluster for Systems Neurology (SyNergy), Munich, Germany; 9 Department of Neurology and Focus Program Translational Neuroscience (FTN), Rhine Main Neuroscience Network (rmn2), University Medical Center of the Johannes Gutenberg University Mainz, Mainz, Germany; 10 NeuroCure Clinical Research Center and Experimental and Clinical Research Center, Charité, University Medicine Berlin and Max Delbrueck Center for Molecular Medicine, Berlin, Germany; 11 Department of Neurology, Philipps-University Marburg, Marburg, Germany; 12 Department of Neurology, University of Leipzig, Leipzig, Germany; 13 Institute of Clinical Neuroimmunology, Ludwig Maximilian University of Munich, Munich, Germany; 14 Department of Neurology, University of Ulm, Ulm, Germany; 15 Clinic of Neurology Dietenbronn, Schwendi, Germany; 16 Department of Neurology, Hannover Medical School, Hannover, Germany; 17 Neurology, Max-Planck-Institute of Psychiatry, Munich, Germany; 18 Neurological Clinic, Sana Kliniken des Landkreises Cham, Cham, Germany; 19 Department of Neurology, Klinikum Augsburg, Augsburg, Germany; 20 Department of Neurology, University of Heidelberg, Heidelberg, Germany; 21 Institut für Neuroimmunologie und Multiple Sklerose, Universitätsklinikum Hamburg-Eppendorf, Hamburg, Germany; 22 Department of Neurology, Neuroimmunological Section, University of Rostock, Rostock, Germany; 23 Department of Neurology, Inselspital Bern, Bern University Hospital and University of Bern, Bern, Switzerland

**Keywords:** Multiple sclerosis, Cognition, Neuropsychology, Cohort study, Longitudinal

## Abstract

**Background:**

Cognitive impairment (CI) affects approximately one-third of the patients with early multiple sclerosis (MS) and clinically isolated syndrome (CIS). Little is known about factors predicting CI and progression after initial diagnosis.

**Methods:**

Neuropsychological screening data from baseline and 1-year follow-up of a prospective multicenter cohort study (NationMS) involving 1123 patients with newly diagnosed MS or CIS were analyzed. Employing linear multilevel models, we investigated whether demographic, clinical and conventional MRI markers at baseline were predictive for CI and longitudinal cognitive changes.

**Results:**

At baseline, 22% of patients had CI (impairment in ≥2 cognitive domains) with highest frequencies and severity in processing speed and executive functions. Demographics (fewer years of academic education, higher age, male sex), clinical (EDSS, depressive symptoms) but no conventional MRI characteristics were linked to baseline CI. At follow-up, only 14% of patients showed CI suggesting effects of retesting. Neither baseline characteristics nor initiation of treatment between baseline and follow-up was able to predict cognitive changes within the follow-up period of 1 year.

**Conclusions:**

Identification of risk factors for short-term cognitive change in newly diagnosed MS or CIS is insufficient using only demographic, clinical and conventional MRI data. Change-sensitive, re-test reliable cognitive tests and more sophisticated predictors need to be employed in future clinical trials and cohort studies of early-stage MS to improve prediction.

**Electronic supplementary material:**

The online version of this article (10.1007/s00415-018-9142-y) contains supplementary material, which is available to authorized users.

## Introduction

Cognitive impairment (CI) and associated neurobehavioral symptoms (e.g., fatigue, depression) are frequent and often highly debilitating in multiple sclerosis (MS) [1]. Particularly cognitive processing speed, executive functions such as working memory capacity as well as verbal and figural episodic memory show a disease-related decline with adverse effects on patient’s vocational status and quality of life [2, 3]. CI has been shown to be present in the earliest disease stages of MS as well as in clinically isolated syndrome (CIS) [4, 5]. Several studies suggest that CI can be present independent of physical disability and that its development and progression is most pronounced during the first years after disease onset [6, 7]. Despite its increasingly recognized clinical relevance for patients with early MS, little is known about risk factors that contribute to CI, its short-term course and a potential progression after initial diagnosis of MS [3, 8, 9]. Associations between clinical disease severity markers (e.g., EDSS, number of relapses, disease duration), conventional MRI parameters of disease burden (e.g., number and/or site of lesions, degree of atrophy) and both severity and profiles of CI have been reported in large cross-sectional cohort studies on a group level [8, 10–12]. However, these associations were less evident in patients with early disease stages [13]. A range of studies have also investigated longitudinally risk factors and prediction of long-term outcome of CI in patients with MS mainly based on clinical and MRI parameters [5, 6, 14–16]. Compatible with results from cross-sectional studies, baseline brain volume [14, 15] and to a lesser degree lesion metrics [6, 16] usually contribute to long-term prediction of CI but predictive abilities were generally low and inconsistent for short-term follow-up periods and early disease stages [5, 14]. Both cross-sectional and longitudinal studies, moreover, display a substantial heterogeneity regarding (i) assessments and definitions of CI, (ii) selection and measurement of predictor variables, (iii) homogeneity of sample characteristics (e.g., disease severity, intake of medication, etc.) and (iv) employed MRI techniques and length of follow-up periods. These methodological issues currently impede an integration and extrapolation of results onto individual cases with newly diagnosed MS [6, 8, 10, 11, 14–17]. In turn, this gap in key-knowledge hinders incorporation of cognitive monitoring into standard clinical care which in turn hampers the development and evaluation of specific programs for the prevention and rehabilitation of CI in MS [1].

Here, we aimed to investigate whether CI and its short-term progression can be effectively predicted by a single marker or combinations of conventional demographic, clinical and MRI parameters that are readily available to clinicians at the time of diagnosing MS. We were further interested in the relative importance of these potential risk factors both for CI as well as for its longitudinal change. To this end, we analyzed cognitive screening data from the German National MS cohort (NationMS) of patients with initial diagnosis of either MS or CIS [18]. We assumed standard sociodemographic data, established clinical markers of MS disease burden and/or conventional MRI parameters at baseline to be predictive for CI. We further analyzed whether changes in cognitive test performance during the first year after diagnosis may be effectively predicted using these baseline parameters.

## Materials and methods

### NationMS cohort study

The German National MS cohort is a prospective longitudinal observational study comprising (a) detailed assessment of patients with first diagnosis of MS or CIS and (b) yearly follow-up assessment with a standardized protocol across 22 centers in Germany. It was approved by the ethics committee of Ruhr-University Bochum (Registration no. 3714-10), and consecutively, by all local committees of the participating centers. All patients provided written informed consent. Inclusion and exclusion criteria as well as assessment plans are laid out in detail elsewhere [18]. In short, inclusion required a recent diagnosis of either CIS or RRMS according to Barkhof [19] or 2005 McDonald [20] criteria, respectively; exclusion criteria implied previous intake of disease-modifying therapies (DMTs), other neurological or psychiatric conditions as well as progressive courses of MS. Assessment involved sociodemographic data, detailed neurological status, medication status regarding DMTs, standardized cranial MRI evaluation regarding signs of disease burden, collection of biomaterial as well as neuropsychological screenings and self-report questionnaires. Datasets from *N* = 1123 patients were included for baseline statistics. Data from *N* = 958 patients were available for follow-up assessment at an average of 12.13 (SD = 1.54) months after baseline.

## Cognitive screening data

### MUSIC: Multiple Sclerosis Inventory for Cognition

The MUSIC is a brief multiple-domain cognitive screening test geared towards rapid assessment of the most frequently impaired cognitive domains in MS [21]. It is widely used as a screening for CI in German-speaking countries and consists of six subtests, in the following order: (1) Word List Learning (number of words learned over two consecutive trials out of a list with 10 words), (2) Interference Word List Learning (number of words learned from a 10 word interference list), (3) Category Fluency Switch Condition (number of correctly associated words within 1 min from two continuously alternating semantic categories), (4) Modified Stroop Task (speed of correctly naming animal silhouettes either in a congruent or incongruent condition with printed animal names on them), (5) Word List Recall (number of correctly recalled words from the initially learned word list after a short delay). For easier inter-test and inter-subject comparisons, individual test scores were *z* standardized based on normative data from *N* = 158 German-speaking healthy young adults as laid out in detail elsewhere [21].

### PASAT: Paced Auditory Serial Addition Test

The PASAT 3-s version is a widely used cognitive screening test in MS tapping into processing speed, divided attention and working memory. PASAT data were extracted from the Multiple Sclerosis Functional Composite (MSFC) [22]. Participants are asked to add numbers in a 1-back-like fashion during a continuous auditory presentation (one number presented every 3 s) and verbally state the correct sums continuously. Outcome measure is the number of correct calculations during a fixed time period. Administration was carried out in accordance with the manual including a preceding training trial and the use of a parallel version at follow-up. Analogous to the MUSIC data, individual PASAT test scores were *z* standardized, stratified for age and education based on normative data from a German sample of *N* = 241 healthy controls [23].

Across all cognitive tests (i.e., subtests of MUSIC and PASAT), a normative *z* score of − 1.645 was used as a cut-off for “impaired performance” as this value approximately represents the 5th percentile rank. Following the criterion put forth by Amato et al., impaired performance in two or more subtests was required to classify individual patients as having CI [6]. Additionally, an unweighted mean *z* score of all cognitive tests was calculated for each patient as a proxy for overall severity of CI.

### Prediction parameters

A priori-considered predictors for CI and longitudinal change are depicted in Table 1. Besides general sociodemographic factors known to influence cognitive status, we examined a range of previously discussed disease-specific risk factors for CI in MS [9]. In total, we considered 17 predictor variables assessed at baseline pertaining to the domains demographics, clinical disease severity markers, MRI ratings of disease burden and self-reports on psychopathology (depressive symptoms and fatigue).

**Table 1:** Baseline predictors and sample characteristics (total *N* = 1123)

Predictor	Available *N*	Mean (SD)
**Demographic characteristics**		
Age, years	1123	34.12 (9.67)
Sex, m:w	1123	348:775
Education, years	1103	14.41 (2.57)
**Clinical characteristics**		
Time since symptom onset, years	1123	0.57 (0.61)
EDSS	1120	1.49 (0.99)
Total number of relapses	958	1.39 (0.62)
Type of disease, RRMS:CIS	1123	622:501
Type of first relapse, mono-:polysymptomatic	1122	831:291
Start of DMT after baseline, yes:no	958	782:176
**MRI characteristics**		
Number of T2 lesions	1117	7.67 (2.21)
Periventricular lesions, yes:no	1123	1081:42
Juxtacortical lesions, yes:no	1123	871:252
Infratentorial lesions, yes:no	1123	666:457
Black holes, yes:no	822	489:333
Visible atrophy, yes:no	877	97:780
**Psychopathological characteristics**		
Depressive symptoms, BDI-II total	1077^a^	7.61 (7.72):185
Fatigue, FSMC total	1073^b^	39.12 (18.21):390

*CI* cognitive impairment, *EDSS* Expanded Disability Status Scale, *RRMS* relapsing–remitting multiple sclerosis, *CIS* clinically isolated syndrome, *DMT* disease-modifying therapy, *BDI* Beck Depression Inventory, *FSMC* Fatigue Scale for Motor and Cognitive functions

^a^*N* = 103 (9.6%) of patients had scores ≥ 19, the clinical cut-off score suggested for indicating an at least mild depressive episode in patients with MS

^b^*N* = 390 (36.3%) of patients had scores ≥ 42, indicating at least mild fatigue

### Statistics

SPSS 25 (IBM Corporation) was used for data preparation and R 3.3.0 (R Foundation, Vienna, Austria) for statistical computations. Descriptive statistics (means and SD as well as frequencies (%) of impaired cases) for baseline and follow-up cognitive data were computed. Change of CI from baseline to follow-up was evaluated using paired *t* tests. Linear multilevel models were applied to predict baseline cognitive test values as well as baseline to follow-up changes in cognitive test values and to control for possible dependency between observations gathered in the same participating center. All predictors were entered into the multiple regression model simultaneously so that co-variance between predictors was controlled for. Models were fitted adopting a Bayesian multilevel approach with the brms package [24] using the probabilistic programming language Stan. For all analyses, a 5% significance level was used and Bonferroni correction was applied within each regression model (that is over 18 regression coefficients per model). Prior to analyses, dichotomous variables (e.g., sex, presence of brain atrophy) were dummy-coded to include them into the regression models. Missing values in predictor variables were imputed by means of 20-fold multiple imputation by chained equations using the mice package [25]. The full analysis is available within the Open Science Framework (https://osf.io/wznca/).

## Results

**Fig. 1:**
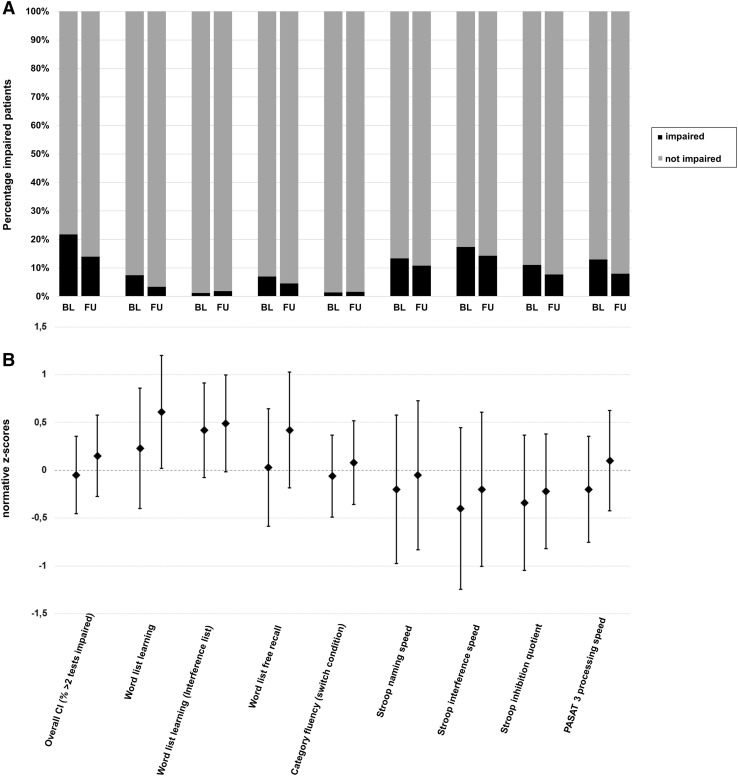
**a** Frequencies of patients with overall CI (≥ 2 tests impaired compared to age- and education-corrected normative data) and of patients with impairments (*z* score <− 1.645) in single cognitive tests for baseline (BL) and follow-up (FU) assessments. **b** Mean normative *z* scores stratified for age and education for overall CI (mean *z* score of all tests) and for each cognitive test separately for baseline (BL, left) and follow-up (FU, right)

Frequencies of patients with and without CI are depicted in Fig. 1a for baseline and follow-up for each cognitive subtest/domain separately. At baseline, a total of 245 (22%) of patients were classified as having CI with the highest frequencies observed in the interference subscore of the Modified Stroop Task (*N* = 185; 17%) of the MUSIC followed by the PASAT (*N* = 135, 12%). Other subtests (e.g., verbal learning and memory) were substantially less frequently impaired. At follow-up, the general profile of relatively frequent impairments in processing speed and executive functions compared to other cognitive domains was similar to baseline. However, substantially less frequent impairments were observed across all tests at follow-up (overall CI in *N* = 120; 14%).

Regarding the severity of deficits, normative *z* scores of baseline cognitive tests and significances of changes from baseline to follow-up are presented in Fig. 1b and Table 2.

**Table 2:** Mean (SD) of unstandardized raw scores and mean normative *z* scores of cognitive tests for baseline, follow-up and longitudinal change

Cognitive test	*N*	Baseline		Follow-up			Longitudinal change	
		Raw mean (SD)	Mean *z* (SD)	*N*	Raw mean (SD)	Mean *z* (SD)	Raw change (SD)	*p*_Bon_
Word list learning (max 20)	1067	15 (3)	0.23 (1.26)	913	16 (3)	0.61 (1.18)	0.81 (2.70)	< 0.001
Interference list (max 10)	1067	6 (2)	0.42 (0.99)	913	6 (2)	0.49 (1.01)	0.13 (1.70)	0.028
Word list free recall (max 10)	1065	6 (2)	0.03 (1.23)	913	7 (2)	0.42 (1.21)	0.74 (2.16)	< 0.001
Category fluency switch	1067	15 (5)	− 0.06 (0.86)	913	15 (5)	0.08 (0.88)	0.77 (4.82)	< 0.001
Stroop naming speed seconds	1066	22 (5)	− 0.20 (1.55)	910	21 (5)	− 0.05 (1.56)	− 0.55 (4.51)	< 0.001
Stroop interference seconds	1066	28.6 (8.3)	− 0.40 (1.69)	910	27.6 (7.9)	− 0.20 (1.61)	− 1.12 (7.81)	< 0.001
Stroop inhibition quotient	1066	7 (6)	− 0.34 (1.42)	910	6 (5)	− 0.22 (1.20)	− 0.57 (6.70)	0.012
MUSIC total score (max 32)	1067	25 (5)	N/A	913	26 (4)	N/A	1.02 (3.87)	< 0.001
PASAT 3 s (max 60)	1038	46 (11)	− 0.20 (1.11)	876	49 (11)	0.10 (1.05)	2.75 (9.10)	< 0.001
Overall CI (mean *z* of all tests)	1038	N/A	− 0.06	921	N/A	0.16	0.21 (0.67)	< 0.001

MUSIC subscores as well as PASAT test scores and corresponding normative *z* scores for baseline and follow-up

*MUSIC* multiple sclerosis inventory for cognition, *Max* maximum raw score, *CI* cognitive impairment, *N/A* not applicable, *PASAT* paced auditory serial addition test, *pBon* Bonferroni-corrected *p* value for paired *t* test of baseline vs. follow-up mean scores

Additionally, spaghetti plots depicting individual cognitive changes from baseline to follow-up can be found in Supplementary Figure 1 for each subtest.

Compared to normative data, the sample’s average overall cognitive ability was not pathological with a mean of all cognitive tests of *z* = − 0.06 at baseline. Compatible with frequency data, processing speed (PASAT, *z* = − 0.20) and executive functions (modified Stroop Test interference seconds, *z* = − 0.40) were the domains with the lowest performances on average. At follow-up, patients performed significantly better on the mean cognitive *z* score (*z* = 0.16 *p* < 0.0001). Likewise, significant gains from baseline to follow-up were observed in the majority of subtests with the exception of the Stroop Inhibition Quotient and the Learning trial of the Interference word list for which no change occurred.

Results of the multilevel linear regression models are presented for the mean *z* score of all cognitive tests representing a proxy for overall CI. Regression coefficients of the model including all predictors for baseline CI are provided in Table 3.

**Table 3:** Regression coefficients for baseline mean cognitive test scores

Coefficient	b	SE	l95% CI	u95% CI	*p*	*p*_Bon_
Intercept	− 0.267	0.162	− 0.585	0.050	0.098	–
**Demographic characteristics**						
Age	− 0.148	0.023	− 0.193	− 0.103	0.000	0.000
Sex (female vs. male)^a^	0.279	0.047	0.186	0.372	0.000	0.000
Education, years	0.188	0.023	0.144	0.233	0.000	0.000
**Clinical characteristics**						
Years since symptom onset	− 0.012	0.026	− 0.063	0.039	0.637	1.000
EDSS	− 0.112	0.025	− 0.161	− 0.064	0.000	0.000
Total number of relapses	0.040	0.047	− 0.052	0.133	0.399	1.000
Type of disease (RRMS vs. CIS)^a^	0.064	0.051	− 0.036	0.166	0.214	1.000
Type of 1st relapse (poly- vs. monosymptomatic)^a^	− 0.046	0.051	− 0.145	0.053	0.368	1.000
Start of DMT after baseline^a^	0.079	0.061	− 0.041	0.200	0.196	1.000
**MRI characteristics**						
Number of T2 lesions	− 0.045	0.022	− 0.088	− 0.002	0.040	0.681
Periventricular lesions present^a^	0.018	0.116	− 0.208	0.247	0.877	1.000
Juxtacortical lesions present^a^	− 0.051	0.054	− 0.156	0.055	0.345	1.000
Infratentorial lesions present^a^	− 0.059	0.044	− 0.144	0.027	0.175	1.000
Black holes present^a^	− 0.013	0.056	− 0.123	0.097	0.823	1.000
Visible atrophy present^a^	− 0.094	0.075	− 0.241	0.049	0.209	1.000
**Psychopathological characteristics**						
Depressive symptoms (BDI-II)	− 0.111	0.031	− 0.170	− 0.050	0.000	0.006
Fatigue (FSMC total)	0.006	0.031	− 0.056	0.067	0.853	1.000

Coefficients in bold indicate a significant influence on the outcome variable (mean *z* of cognitive test performance at baseline). Negative regression coefficients indicate that larger values in the predictor have a negative influence on the outcome variable and vice versa

*b* regression coefficient, *SE* standard error, *l95% CI* lower bound of the 95% credible interval, *u95% CI* upper bound of the 95% credible interval, *p* uncorrected two-sided *p* value, *pBon* Bonferroni corrected two-sided *p* value

^a^Dichotomous variables that have been dummy-coded prior to analysis

The proportion of variance explained by this model was *R*^2^ = 0.27 when including the variance explained by the participating center and *R*^2^ = 0.21 without it. The predictors that remained significant after Bonferroni correction were age (“more CI in older patients”), years of education (“more CI in patients with fewer years of academic education”), EDSS score (“more CI in patients with higher EDSS”), BDI-II score (“more CI in patients with more self-reported depressive symptoms”), and sex (“more CI in males”). Other MS-specific clinical or MRI characteristics did not significantly contribute to the prediction of baseline CI. Regression coefficients of the model including all predictors for the baseline to follow-up changes in cognitive test scores are provided in Table 4.

**Table 4:** Regression coefficients for baseline to follow-up changes in mean cognitive test scores

Coefficient	b	SE	l95% CI	u95% CI	*p*	*p*_Bon_
Intercept	− 0.273	0.228	− 0.723	0.174	0.234	–
**Demographic characteristics**						
Age	− 0.073	0.035	− 0.141	− 0.005	0.034	0.580
Sex (female vs. male)^a^	0.141	0.072	0.001	0.283	0.049	0.826
Education (years)	− 0.031	0.035	− 0.099	0.036	0.372	1.000
**Clinical characteristics**						
Years since symptom onset	0.044	0.040	− 0.034	0.124	0.266	1.000
EDSS	0.086	0.037	0.013	0.157	0.022	0.370
Total number of relapses	− 0.045	0.066	− 0.176	0.085	0.497	1.000
Type of disease (RRMS vs. CIS)^a^	− 0.037	0.079	− 0.192	0.117	0.642	1.000
Type of 1st relapse (poly- vs. monosymptomatic)^a^	0.106	0.077	− 0.044	0.258	0.173	1.000
Start of DMT after baseline ^a^	− 0.125	0.088	− 0.299	0.048	0.158	1.000
**MRI characteristics**						
Number of T2 lesions	− 0.006	0.034	− 0.072	0.059	0.850	1.000
Periventricular lesions present^a^	0.211	0.176	− 0.134	0.553	0.231	1.000
Juxtacortical lesions present^a^	0.149	0.083	− 0.015	0.311	0.076	1.000
Infratentorial lesions present^a^	0.072	0.068	− 0.061	0.204	0.294	1.000
Black holes present^a^	− 0.114	0.087	− 0.286	0.055	0.188	1.000
Visible atrophy present^a^	0.124	0.113	− 0.093	0.348	0.271	1.000
**Psychopathological characteristics**						
Depressive symptoms (BDI-II)	0.080	0.051	− 0.020	0.181	0.118	1.000
Fatigue (FSMC total)	− 0.106	0.053	− 0.212	− 0.003	0.042	0.715

Coefficients in bold indicate a significant influence on the outcome variable (mean z of baseline to follow-up change of cognitive test performance). Negative regression coefficients indicate that a larger value in the predictor has a negative influence on the outcome variable and vice versa

*b* regression coefficient, *SE* standard error, *l95% CI* lower bound of the 95% credible interval, *u95% CI* upper bound of the 95% credible interval, *p* uncorrected two-sided *p* value, *pBon* Bonferroni corrected two-sided *p* value

^a^Dichotomous variables that have been dummy coded prior to analysis

No predictor remained significant after Bonferroni correction indicating that longitudinal cognitive change could neither be effectively predicted by the considered baseline variables nor the additional variable of DMT initiation after baseline (yes vs. no). The proportion of variance explained was *R*^2^ = 0.06 when including the variance explained by participating center and *R*^2^ = 0.05 without it. Likewise, results for each separate cognitive subtest were non-significant regarding the prediction of cognitive change from baseline to 1-year follow-up. These and other additional analyses are provided as supplementary material on https://osf.io/wznca/.

## Discussion

Despite the increasingly recognized burden of CI in MS, little is known about an increased individual risk for CI after initial diagnosis of MS, hampering research on early prevention and treatment. In the current study, we aimed to characterize CI and identify risk factors for its severity and short-term course in a large, clinically homogeneous cohort of patients with first diagnosis of MS or CIS. To this end, neuropsychological screening data from *N* = 1123 patients enrolled in the multicentric German National MS cohort study were analyzed. We used linear multilevel regression models to predict CI and the short-term progression of CI from conventional MRI characteristics and other clinical and demographic parameters that are usually accessible to clinicians at the time of diagnosis.

### Frequency, severity and profile of CI

Adopting conventional criteria of overall CI, we found 22% of patients to be impaired at baseline, with largest deficits in subtests for processing speed and executive function and lowest impairments in verbal learning and memory. The result of a relatively larger impairment in attention and processing speed as compared to other cognitive domains is well in line with previous studies on the cognitive profile of patients with early MS [6, 8] and CIS [1, 3]. Overall frequency and mean severity of CI was lower in our sample than commonly reported: the majority of previous studies found approximately one-third of patients with CI in early MS or CIS [3, 6, 7], although reported frequencies range from < 15 to > 50% [5, 26]. One explanation for this discrepancy may be that the current sample is unique in terms of a homogeneous sample in a very early disease stage with a median disease duration of only 0.33 years [18]. Compensatory mechanisms such as cognitive reserve may attenuate direct measurability of CI specifically in young patients with low overall disease burden and high formal education resulting in lower frequencies [17]. Hence, patients with larger cognitive reserve capacity may be able to compensate for brain pathology despite suffering from clinically relevant CI [13]. An additional explanation for our finding of a lower prevalence of CI in patients with early MS and CIS may be that the employed screening tests are less sensitive to detect CI in these early disease stages that might extend beyond executive and speed-related domains. Reports on the prevalence of CI in MS depends on (a) the employed tests (e.g., screening tests only or extensive test batteries), (b) the formal definition of CI (e.g., one or two standard deviations below the norm; comparison to a control group), and (c) the composition of the sample (e.g., patients with progressive MS show a different degree of CI than patients with early MS or CIS [27]). Internationally accepted standards regarding screening for CI have been proposed in terms of the Brief International Cognitive Assessment in MS (BICAMS battery) and may allow a higher sensitivity to detect relevant CI in MS throughout the different disease stages [28]. For instance, the Symbol Digit Modalities Test (SDMT) has been shown to be a more reliable, and sensitive measure of cognitive processing speed than the PASAT employed in this study [29, 30]. More specific cognitive functions like calculation skills may as well influence individual PASAT results. Thus, while the Modified Stroop Task of the MUSIC was able to detect early deficits in processing speed and executive function, the single-trial ten-item list might be insufficient to reveal subtle memory changes that might unfold in a multiple-trial learning-paradigm.

### Predictors of CI and its progression

We found baseline CI to be significantly associated with three general demographic characteristics: male sex, fewer years of education and higher age. These factors have previously been linked to lower (verbal-)cognitive test performance in healthy adults suggesting influences that are not specific to MS or CIS but may, nevertheless, be of clinical importance for the interpretation of MS patients’ test performances [31, 32]. Considering MS-specific clinical characteristics, only EDSS (a marker for mainly physical disease burden) and severity of depressive symptoms (BDI-II) were associated with severity of CI at baseline. These results are in line with previous evidence from large patient samples finding that higher EDSS and depressive symptoms negatively influence cognitive status [10, 27, 33]. Surprisingly, none of the conventional MRI (e.g., visual inspection of atrophy, number of T2 lesions) or other clinical predictors (e.g., type of disease CIS/RRMS, total number of relapses) that have previously been directly linked to CI and its long-term course contributed to prediction. This result may again cast doubts on the sensitivity of the employed screening tests to reliably detect CI in early disease stages. In the current sample, however, brain pathology and disease severity were also homogeneously low and relationships between CI and conventional markers for structural brain damage may be generally weak in early MS, even when using more sophisticated neuropsychological assessments. In a recent large cohort study, lack of association between brain pathology (as measured by voxel-based morphometry) and performance in the BICAMS test battery was termed a “clinico-radiological paradox” and attributed to both, stronger compensatory mechanisms (e.g., cognitive reserve) and a statistical restriction of range within a homogeneous sample of patients in early disease stages [13]. Despite the large sample size and the numerous considered clinical, demographic and conventional MRI baseline parameters as well as the variable of DMT initation after baseline, the longitudinal change of cognition over the course of 1 year could not be sufficiently predicted. One explanation may be that, for instance, for the considered MRI parameters and DMT initiation, the categorization was too broad (e.g., dichotomization DMT start yes vs. no, visible MRI atrophy yes vs. no). On the other hand, the follow-up interval of 1 year may be too short to detect clinically relevant changes. However, significant gains in cognitive performance were observed in the majority of patients and in most cognitive subtests. This strongly suggests that test performances in both, MUSIC and PASAT, were substantially influenced by practice effects, potentially masking clinically relevant longitudinal changes after 1 year. A recent review has estimated the average effect size of cognitive retesting in a 12-month interval to be as high as 0.25 while some standard neuropsychological tests reached effect sizes of 0.73 [34]. Likewise in patients with MS, carryover effects from one testing session to another is a frequent problem in longitudinal test designs and common to a range of neuropsychological tests including the PASAT and to lesser degrees also the SDMT [29, 35, 36]. Although alternate test versions matched for difficulty and modern regression-based normative data (including estimates for retesting effect-sizes) may attenuate the influence of practice effects, few standardized cognitive tests employed in testing patients with early MS provide these features. Moreover, despite the use of an alternate version in the PASAT in this study, patients on average performed significantly better at follow-up, highlighting a likely influence of familiarity that is not dependent on the particular stimuli. Practice effects may endure for approximately 1 year after a baseline assessment and are most pronounced between the first and second evaluations [37, 38]. This is, particularly, true for tests assessing memory, learning and executive functions while visuo-perceptive tasks are less prone to practice effects [39]. Hence, the difference of some cognitive tests in their resilience against practice effects has to be considered more rigorously when planning re-evaluation schedules. Moreover, additional cognitive testing (performed outside of the study or by patient self-assessment and training) needs to be controlled for.

## Conclusions

In patients first diagnosed with MS or CIS, demographic characteristics (male sex, higher age, lower education) as well as more severe depressive symptoms (BDI-II) and higher physical disability (EDSS) are significantly associated with severity of CI. In patients with these characteristics, neuropsychological monitoring and potentially cognitive rehabilitation should be considered. No other disease-specific clinical or conventional MRI parameters from clinical routine were significantly related to the presence of CI in this large cohort of patients in earliest disease stages. Moreover, longitudinal prediction of short-term cognitive change over the course of 1 year was insufficient despite the large number of patients and the inclusion of numerous conventional yet disease-specific and previously discussed predictor variables. These findings indicate that three branches of research are highly needed to increase our understanding of CI, its clinical relevance and its risk factors in early MS to blaze the trail for early interventions: (1) establishment and evidence-based proof of sensitive and change-sensitive cognitive outcome parameters providing free-to-use longitudinal normative data. (2) Evidence that these assessments are able to detect disease-specific and clinically relevant CI (i.e., by validation with patient-centered outcomes) from the earliest to advanced disease stages. (3) Improving the prediction of these measurements by the development of refined clinical scales and standardized automation of MRI parameters for use in clinical routine [40].

## Electronic supplementary material

Below is the link to the electronic supplementary material.

**Suppl. Fig. 1**: Spaghetti-plots indicating individual cognitive changes from baseline to follow-up for overall CI (mean *z* score of all tests) and for each cognitive test separately. (TIFF 11390 KB)
