# Supplementary figures and images for: Can we predict cognitive decline after initial diagnosis of multiple sclerosis? Results from the German National early MS cohort (KKNMS)
Source: J Neurol. 2018 Dec 4;266(2):386–97. doi: 10.1007/s00415-018-9142-y (PMC6373354; doi:10.1007/s00415-018-9142-y)

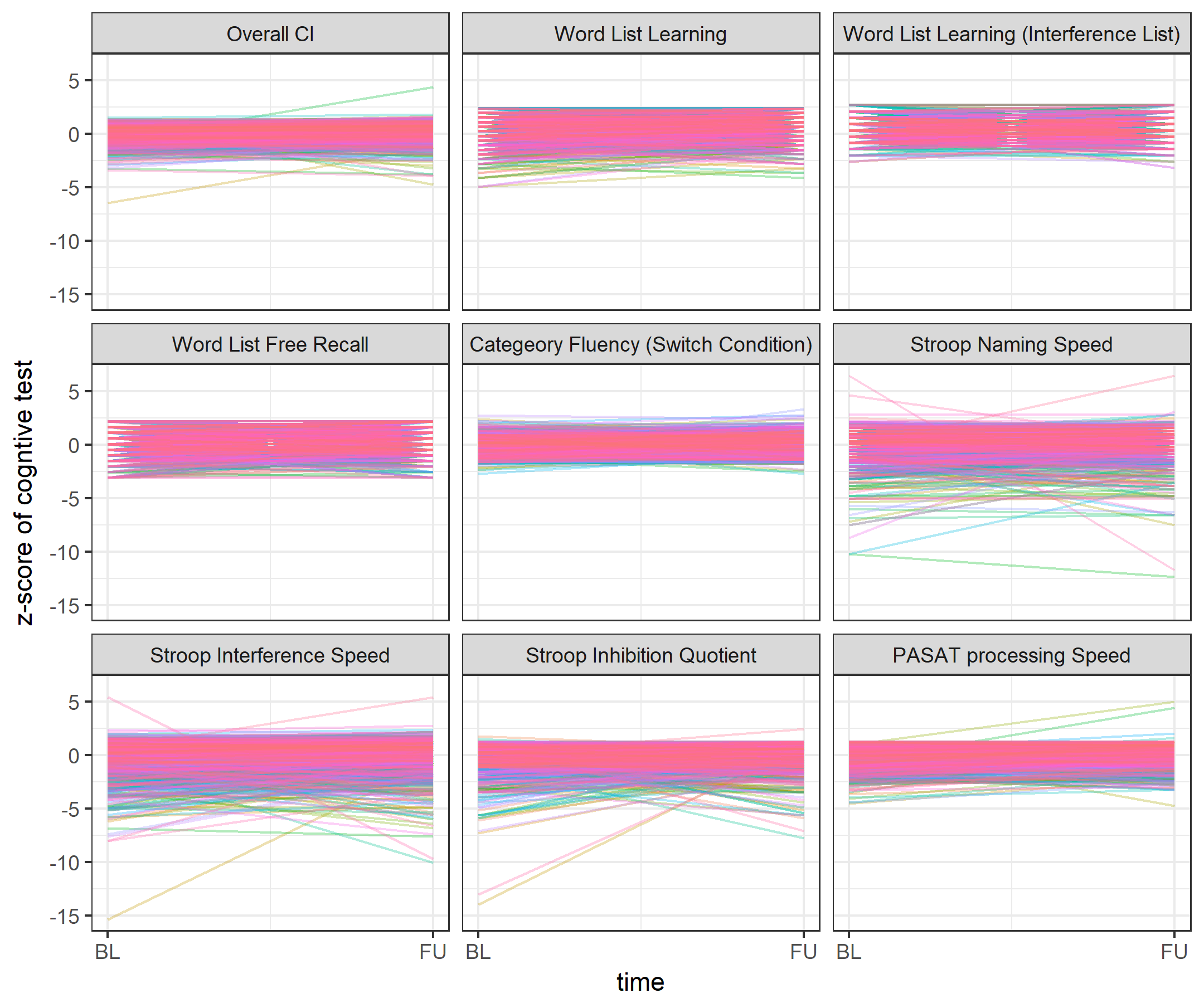

Supplement: Supplementary file 1 — Suppl. Fig. 1: Spaghetti-plots indicating individual cognitive changes from baseline to follow-up for overall CI (mean z score of all tests) and for each cognitive test separately. (TIFF 11390 KB) [file 415_2018_9142_MOESM1_ESM.tiff]
